# Who Will Shape the New World Order?

**DOI:** 10.1007/s10272-022-1054-5

**Published:** 2022-06-07

**Authors:** Karl Aiginger

**Affiliations:** c/o ÖGfE, Sekretariat Maxine Amstätter, Policy Crossover Center: Vienna - Europe, Rotenhausgasse 6, 1090 Vienna, Austria

**Keywords:** E61, F00, P40

## Abstract

The economic consequences of the war could potentially be used to postpone climate policy, energy saving and the shift to renewable energy. This includes investments in atomic energy — perhaps in smaller plants — as there is still no satisfactory solution for the storage of nuclear waste.

Globalisation has brought large benefits: It has reduced poverty at an unprecedented pace, opened up new possibilities, and led to more diverse and cheaper products. But it has also led to problems such as greater inequality in rich countries due to job losses in old industrial regions, a necessity for change in education and occupations, and international monopolistic firms exerting a great influence on politics. The division of labour has led to new dependencies, with firms producing intermediate products for distant customers. Technologies have changed, requiring new resources and rare metals. Supply chains have become longer, increasing the danger of disruptions.

At the same time, recent developments have led to a call for a new type of globalisation requiring better rules that force firms to take responsibility for their input chains. It has become clear that free riding on climate and social policy issues should be prevented, whether through carbon border taxes or emissions trading. International courts should be less dominated by rich countries and should follow World Trade Organization supervision. This has been called "responsible globalisation" or the "end-of-fast-track globalisation".

The war in Ukraine has created a new context and made rapid changes necessary, especially if the old problems are not to be exacerbated. As with any radical new situation, it can either become a turbo mechanism for reform or an excuse for delaying the necessary changes that are unpopular among lobbies and vested interests. We argue that this presents an opportunity that should be taken, and the temptation to return to past policy should be avoided.

## Globalisation will change

The share of people living in absolute poverty as defined by a monetary boundary of one or two dollars' income per day has declined rapidly – it was halved much faster than envisaged by the Millennium Development Goals. This initially happened primarily in Asia, then later in other emerging countries, and most recently – limited by large population growth and increasing droughts – in Africa.

Growth in poor countries has been higher than in rich ones, with the US maintaining its lead in GDP per capita. Many countries shifted production from raw materials to industry, which led to low-or-middle-income traps and cases of "Dutch disease" for those countries that did not use the resources provided by raw material exports to upgrade skills and climb the quality ladder.

Higher income has not led to reduced inequality within nations; inequality returns in ever new forms, if it is not addressed by political policy. Regional inequalities increased as a result of rapid globalisation, since old industrial regions felt left behind, creating fertile ground for populist or nationalist movements claiming past times were much better. Firms have been closed in old industrial areas, and these regions have not succeeded in attracting new ones. Migrants or refugees have been made responsible for problems (e.g. in Hungary, France, the UK and the US).

Climate change was acknowledged as an important problem but did not receive enough attention in terms of policy. Developing countries had no chance to limit emissions due to a lack of resources and technologies. Rich countries claimed poorer countries should not have a free pass with regard to ecological concerns, as they were generating greater emissions per population or output. While the latter is correct, the assertion did not take into consideration that the industrialised countries had the technology for change and had used up the largest share in the storage capacity of our planet in the past.

The rewards of globalisation have often been skimmed off by oligarchs, rather than used to increase wages, which have, for example, stagnated in the US over the past 30 years. High-income earners and the financially independent have been able to escape taxation by shifting headquarters and profits into tax shelters. Strong firms have been able to demand free access to markets through international investment compacts and courts. Reducing child labour and racial or gender inequality has not always been successful, with corrupt and autocratic governments

**Karl Aiginger**, Policy Crossover Center Vienna-Europe; and WU Vienna University of Economics and Business, Austria.

seeking to buy weapons and rich countries competing to provide them. Meanwhile, regional conflicts have persisted and the streams of refugees, initially directed towards neighbouring countries, have remained substantial.

The benefits of globalisation have been measured by old GDP, though this was never a measure of welfare. Switching to other narrow or broader measures, such as life expectancy or Sustainable Development Goals, revealed many benefits. It has become clear that democracy does not automatically deepen with higher income or welfare. And freedom can take on different forms, while still not being guaranteed by higher incomes and open trade. Rich countries always think that they are the leaders in democracy and freedom, even if old structures and racial conflicts remain. Leading policy groups can do a lot to maintain their power. The reduction of early deaths and the prolongation of life expectancy, including healthy life terms, continues to be a benefit of globalisation. Adding peace and free movement is also all-important.

Globalisation needs to be accompanied by a forward-looking and responsible policy. In developing countries, this may mean using rewards to upgrade education, basic skills and innovation. Medium-income countries have to learn from foreign technologies and invest in vocational and high schools. High-income countries must invest in international and open universities, and industrial policy has to change to include the definition of lead industries and digitisation. Improving education is imperative in all groups.

## The dichotomous impact of war on Europe

If we leave out the direct impact of the war in Ukraine on human suffering, we see that several options exist for countries not directly involved in the conflict.

Most people and nations have meanwhile realised that climate change is real, that it is human made, that fossil energy sources played an important role and that global warming is dangerous. Following the Paris Agreement and last year's UN Climate Change Conference in Glasgow, many nations committed to ambitious goals that offer a last chance to stop global warming at a time when deaths due to heat waves and erratic weather are already playing a larger role than deaths caused by traffic accidents. The negative consequences of climate change are furthermore unevenly divided between nations that have contributed significantly to it in the past and nations that have had fewer possibilities to stop it. The goals set for achieving climate neutrality by mid-century would require action. Due to the new problems created by the war, the climate goals have been downgraded on the agenda or postponed. Peace is all-important but is used as a justification for finding new sources of fossil energy and further exploring the sea and other regions to find liquid natural gas, even if this in turn requires new resources, emissions and long transport chains. Expenditures on limiting climate change have been delayed, while new importance has been placed on current production along old paths.

We knew that we could significantly save energy and waste, but we now realise the extent to which many nations, including Germany and Austria, still rely on imports of gas and oil, much of which comes from Russia or its partners. We knew that we needed to have reserves, since clean energy is only discontinuously available and to a degree unstable, but then had to acknowledge that the storage capacities for gas were not even half-replete (and were partly owned by Gazprom).

We knew that external safety could to an extent be ensured by a peaceful agenda in Europe, but that NATO and its weapons and forces would ultimately be responsible for security in case of an aggression or conflict. Many European countries currently feel safer as members of NATO, with Finland and Sweden now applying for membership. Other countries are trying to increase their defence expenditures, even if existing expenditures are oriented towards past conflicts, instead of helping prevent humanitarian and ecological emergencies.

### Europe may react by postponing necessary changes …

The economic consequences of the war could potentially be used to postpone climate policy, energy saving and the shift to renewable energy. This includes investments in atomic energy – perhaps in smaller plants – as there is still no satisfactory solution for the storage of nuclear waste.

This scenario can be observed in many countries. Several mainstream parties have proposed cutting taxes on energy and populist parties have been able to win elections with strongmen. But people seem generally discontent, as the large shares of the population voting for both right-wing and left-wing parties demonstrate – for example, in France where these parties together have been stronger than that of Macron and the old mainstream parties.

European countries are longing for new providers of fossil energy, whether this means buying more oil from Arab countries, Iran or Turkey, or more liquefied gas from the US, provided additional terminals are created in Europe. Some have deplored the idea of horizontal drilling in Europe due to its environmental costs. There is an imminent concern that Russia and Gazprom will cease to fulfil their contracts.

Europe has the largest public sector relative to GDP and very high taxes, but also larger subsidies for fossil energy sources than for renewables. A proposal to cut taxes makes sense, but this should not begin with taxes on energy, which are set at a level far below their external costs, and planned steps to tax carbon emissions should not be delayed, even amid rising inflation.

### …or become more ambitious

The better alternative would be to accelerate overall energy and resource saving, while shifting further energy needs to renewables. We know that energy efficiency is very different e.g. it is three times higher in Switzerland than it is in the US. The shift to renewable energy could happen at a much faster pace. Southern European countries can cover a large portion of their demand through wind and solar energy but may not have excelled in the field of renewable technologies. France has a better greenhouse gas balance, but this is mainly due to its use of nuclear energy. New European funds for investment and resilience should be used much more intensively to shift demand to non-fossil energies, public transportation, electric cars or cycling. Power for ships and planes must be taxed, and innovations for better fuels are around the corner. Short flights of up to 500 kilometres are inefficient. Public expenditures should be redistributed from subsidising energy consumption to improving buildings and making towns greener, while taxes should be reduced on low incomes.

Europe has taken some steps in this direction with its Green Deal and in setting earlier targets for climate neutrality. The Fit for 55 package looks much better than past plans, but the goals still lie far below the change required to meet the Paris target (a 55% reduction over forty years amounts to not much more than one percent annually). That any further compromises – thought as necessary because of a possible end to gas deliveries from Russia – would lead Europe further away from the Paris path is evident, but this has been forgotten by both the mainstream and the populist parties.

## Towards a new world order

It is no consolation that Europe is not the only region taking steps in the wrong direction. Negative externalities – domestically and around the world – are priced much lower than necessary, with some politicians calling for a return to old solutions. Ecological policy is not looking for synergies with social and health policy. Even in countries that have a long tradition of democracy, the realisation of democracy is far from perfect and populistic inroads are frequent (Aiginger and Colcuc, 2022).

### Who will act?

The old world order broke down after the demise of the Soviet Union. The unipolar moment for the US as a leader was neither accepted by other countries nor very successful, since problems and demand are very different and the US is not ready to take other preferences into account. China has invested significantly domestically and around the world, always from a perspective of what was best for China. It is attempting to change world institutions and become the first socialist superpower (with ambitions to expand its territory, such as in Taiwan and the Solomon Islands).

One possibility would be a closer cooperation between Russia and China. Russia may wish to export resources eastwards, if sanctions and isolation persist after the Ukraine war. China is reluctant to cooperate, fearing it could be included in the sanctions, but will accept higher bilateral trade if new resources help its economy.

A closer cooperation between Europe and the US is possible in theory, but the US is focusing more on the Indo-Pacific region, perhaps in the form of an extended AUKUS bloc (Australia, the UK and the US). Meanwhile, the UK is seeking to play as central a role as it did under the Commonwealth, which will not be easy after Brexit.

India does not wish to cooperate all that closely with China, but has not yet decided how to proceed. It has not criticised Russia's war with Ukraine, it continues to have problems with Pakistan, and it must manage an enormous population striving for work and higher welfare under a nationalist government.

Europe has been divided internally for a long time, with differences between northern and southern EU countries as well as older and newer members. And there has been tension between the bloc and the countries that wish to become members, but do not fulfil all the requirements. The EU has not been able to agree on border control policies or quotas for refugees, and the accession process for the Western Balkan countries has been too slow. Planning a step-by-step future enlargement would be a possibility (Wieser et al., 2022).

The war in Ukraine could be a game changer. Europe has reacted swiftly and to an extent in a more united way than ever before. The member countries quickly agreed that Russia was the aggressor, and that NATO could not interfere directly, while European countries could provide either weapons or humanitarian aid. Europe's borders have been open to refugees from Ukraine, who are free to travel wherever they wish and are quickly welcomed in childcare facilities, schools and the work force. This also signals a change in overall attitude towards migrants, which could place less of an emphasis on the countries of origin in the future.

## Do we need a leader and, if so, who could assume the role?

Some of the current problems will subside in the wake of the COVID-19 pandemic crisis and the war in Ukraine. But both of these crises have only accelerated the reshaping of globalisation and the formation of a new world order.

Europe has assumed a central role with regard to the Ukraine war, imposing sanctions on Russia and providing assistance to Ukraine, which was previously not considered possible, due to Europe's smaller size and lack of internal consensus. However, this new role is indeed necessary, following the retreat of the US from its position as the sole remaining superpower often engaging in conflicts without long-lasting reason and China still focusing on its self-centred agenda, including a zero-COVID-19 strategy. European countries still have differing positions with respect to Russia (Serbia and Hungary have, for example, refrained from criticising the invasion of Ukraine). An EU offer of fast-tracked membership to Ukraine – perhaps in phases – would accelerate the accession process for the Western Balkans (Wieser et al., 2022).

Europe can play a much stronger role in the new world order. It currently leads in terms of broader welfare measures, life expectancy, ecological sustainability and most Sustainable Development Goals (Aiginger and Colcuc, 2022). Russia has long disqualified itself. The US has dropped out of advances in climate policy, shrunk its military role and hesitated in deciding on its future course, partly due to midterm elections and internal divisions. China has hesitated to criticise Russia's aggression in Ukraine, hoping the war will increase its chances of obtaining oil from Russia and distract attention from its territorial policies regarding Taiwan, the South China Sea or the Artic.

However, Europe must keep an eye on potential new world partners and prioritise better cooperation among current and future members. It should invest resources in fighting disease and preventing environmental damage in Africa as well as in neighbouring countries. It must focus on forward-looking policies, also with regard to inflation or migration, and it is absolutely unacceptable to postpone necessary changes when a new problem occurs. Large and often dysfunctional taxes and government systems must be addressed.

Putin's war constitutes a break. Europe should accelerate its active path of decarbonisation and partnerships with neighbours, rather than return to anachronistic technologies, including the import and use of fossil fuels. The public sector can navigate this future path by imposing taxes on emissions and externalities while rewarding innovative solutions, training for young people and retraining. Steps can include a different kind of cooperation with Russia after the war in Ukraine, for example, in the form of investments like those outlined in the Marshall Plan after World War II that fostered peace, reconciliation and reconstruction. This would strongly position Europe in a new world order, given its strength in many aspects that are necessary for increasing welfare.
